# Establishment and characterisation of patient-derived xenografts as paraclinical models for gastric cancer

**DOI:** 10.1038/srep22172

**Published:** 2016-03-01

**Authors:** Yoon Young Choi, Jae Eun Lee, Hyunki Kim, Moon Hee Sim, Ka-Kyung Kim, Gunho Lee, Hyoung-Il Kim, Ji Yeong An, Woo Jin Hyung, Choong-Bai Kim, Sung Hoon Noh, Sangwoo Kim, Jae-Ho Cheong

**Affiliations:** 1 Department of Surgery, Yonsei University College of Medicine, Seoul, Korea; 2 Yonsei Biomedical Research Institute, Yonsei University College of Medicine, Seoul, Korea; 3 Department of Pathology, Yonsei University College of Medicine, Seoul, Korea; 4 Severance Biomedical Science Institute, Yonsei University College of Medicine, Seoul, Korea; 5 Brain Korea 21 PLUS Project for Medical Science, Yonsei University College of Medicine, Seoul, Korea; 6 Department of Biochemistry & Molecular Biology, Yonsei University College of Medicine, Seoul, Korea; 7 Department of Surgery, Samsung Medical Center, Sungkyunkwan University School of Medicine, Seoul 06351, Korea; 8 Open NBI Convergence Technology Research Laboratory of Department of Surgery, Yonsei University College of Medicine, Seoul, Korea

## Abstract

The patient-derived xenograft (PDX) model is emerging as a promising translational platform to duplicate the characteristics of tumours. However, few studies have reported detailed histological and genomic analyses for model fidelity and for factors affecting successful model establishment of gastric cancer. Here, we generated PDX tumours surgically-derived from 62 gastric cancer patients. Fifteen PDX models were successfully established (24.2%, 15/62) and passaged to maintain tumours in immune-compromised mice. Diffuse type and low tumour cell percentage were negatively correlated with success rates (*p* = 0.005 and *p* = 0.025, respectively), while reducing *ex vivo* and overall procedure times were positively correlated with success rates (*p* = 0.003 and *p* = 0.01, respectively). The histology and genetic characteristics of PDX tumour models were stable over subsequent passages. Lymphoma transformation occurred in five cases (33.3%, 5/15), and all were in the NOG mouse, with none in the nude mouse. Together, the present study identified Lauren classification, tumour cell percentages, and *ex vivo* times along with overall procedure times, as key determinants for successful PDX engraftment. Furthermore, genetic and histological characteristics were highly consistent between primary and PDX tumours, which provide realistic paraclinical models, enabling personalised development of treatment options for gastric cancer.

Gastric cancer is one of the most important health problems in the world; it is the 5^th^ most commonly diagnosed cancer and the 3^rd^ most common cause of death from cancer^1^. For advanced gastric cancer, additional treatment followed by surgical resection is mandatory, but the clinical response to standard chemotherapy varies among patients, and biologically-targeted therapeutics are rarely utilized^2^,^3^,^4^,^5^,^6^,^7^. These heterogeneous treatment outcomes and lack of clinically usable targeted therapeutics represent urgent clinical needs, and emphasize the necessity of developing effective personalized treatments for patients with gastric cancer, based on the tumour’s molecular and genetic characteristics. To achieve effective cancer therapies, more realistic models based on the biological characteristics of individual patients are needed to predict the response to therapy. Traditionally, cancer cell lines and xenograft models derived from the established cell lines have been used for drug screening, to characterise the biology of specific tumours and to identify optimal drug candidates for therapy. Despite several advantages, the monodimensionality of cells grown in culture, and xenograft models that have adapted to growth in artificial culture conditions, largely differ from primary tumours^8^. They poorly represent the heterogeneity and genetic features of patient tumours. Consequently, predictive values for clinical outcomes, based upon these systems, have been largely unsatisfactory^9^,^10^.

Patient-derived xenograft (PDX) tumours, which are xenograft models developed by transplanting human tumours into immune-compromised mice, have been suggested as a more realistic preclinical cancer model^11^,^12^. PDX tumours retain the majority of key genes expressed in primary tumours^8^,^13^, and they correlate well with clinical responses to chemotherapy^14^,^15^. Recently, there has been renewed interest in establishment of PDX models for various cancers by investigators in academic and pharmaceutical research, to improve the development of effective therapeutics^16^,^17^,^18^. Despite the potential importance of the PDX model for cancer research and clinical translation, few studies have reported histological and genomic fidelities of the model systems, and few reports have identified factors correlated with engraftment success of gastric PDX tumours. The purpose of the present study was therefore to characterise the histological and genomic fidelities of gastric cancer PDX models and identify the factors related to the successful engraftment in mice.

## Results

### Baseline characteristics

To establish PDX tumours, a total of 161 mice (75 nude mice and 86 NOG mice) were used as recipients of tumours from 62 gastric cancer patients. The baseline characteristics of donor patients are summarized in Supplementary Table S1. The median patient age was 61 years, and 77.4% (48 out of 62) were male. Thirty-eight (61.3%) patients were diagnosed as stage I/II, and the other patients were diagnosed as stage III/IV (37.1%). There were 30 patients (48.4%) with lymph node metastasis. Thirty-six (58.1%) patients had undifferentiated histology, while 19 (30.6%) and 5 (8.1%) patients had differentiated histology, with carcinomas with lymphoid stroma or mixed histology, respectively. There were 32 cases (51.6%) of intestinal tumours, 18 cases (29.0%) of diffuse tumours, and 10 cases (16.1%) of mixed or indeterminate tumours.

### Establishment of PDX models

PDX tumours were initially generated in F1 mice, then implanted into F2 mice in 15 cases (24.2%, 15 out of 62). Table 1 summarizes the characteristics of these successful cases. The histological types were poorly differentiated (n = 5), moderately differentiated (n = 6), mucinous (n = 2), moderately differentiated with signet ring cells (n = 1), and mixed adeno-neuroendocrine (n = 1) carcinomas. Using the Lauren classification^19^, 12 tumours were intestinal, two tumours were indeterminate, and one tumour was mixed. The implantation locations for F1 in successful engraftment cases were subcutaneous of the flank in 14 mice, and the subrenal capsule in one mouse. The recipient mouse strains were six nude mice and nine NOG mice. The median percentage of tumour cells in the tissue fragments was 60% (5–90%), and the median elapsed time for engraftment in F1 animals was 94 days (44–160 days). The details of the growth rate in F1 animals are shown in Supplementary Fig. S1. There was no difference between the nude and NOG mice regarding growth rates and elapsed time.

### Factors related to successful engraftment of PDX tumours

To investigate potential factors related to the success of PDX tumours, we classified the parameters into three groups: patient characteristics, pathological tumour characteristics, and experimental parameters. There were no statistically significant parameters correlated with success of PDX tumours in patient characteristics such as age, gender, co-morbidity, history of smoking and alcohol, and no statistically significant parameters correlated with the success of PDX tumours in laboratory parameters, including tumour markers such as carcinoembryonic antigen (CEA) and carbohydrate antigen 19–9 (CA19–9) (Supplementary Table S2). Regarding tumour pathological characteristics, the location, gross type, tumour aggressiveness such as tumour size, invasion depth, lymph node metastasis, and TNM stage, and preoperative chemotherapy were not correlated with engraftment success (Table 2). There were no engraftment successes among 18 cases of the diffuse type of gastric cancer, while PDX tumours were established in 12 (37.5%) out of 32 cases of intestinal tumours, and 3 (30%) out of 10 cases of mixed or indeterminate type tumours (*p* = 0.005). In addition, higher tumour cell percentages in the representative tissues were correlated with higher success rates (*p* = 0.025).

Among the experimental parameters, *ex vivo* times were shorter in successful cases than unsuccessful cases (median time was 75 minutes for successful cases versus 135 minutes for unsuccessful cases, *p* = 0.003, Table 3). In addition, a short overall procedure time correlated with engraftment success (123 minutes for successful engraftments versus 167 minutes for unsuccessful engraftments, *p* = 0.01). When multiple pieces were implanted in a single animal, the success rate was higher (38.9%) than for implantation of a single piece (18.2%), but this difference was not statistically significant (*p* = 0.108). Other experimental parameters, such as the mouse strain, implantation site, or using Matrigel were not correlated with engraftment success (they were analysed based on the number of mice). The success rate was 8.0% for nude mice (6 out of 75) and 10.5% for NOG mice (9 out of 86). Furthermore, 10.7% of subcutaneous implantations were successful (14 out of 131), while only 3.3% of subrenal capsule implantations were successful (1 out of 30), and Matrigel-embedded tumours did not improve the engraftment success (11.1% without Matrigel versus 13.6% with Matrigel, *p > *0.999, Supplementary Table S3).

### Histological and genomic features of PDX tumours

**Figure 1:**
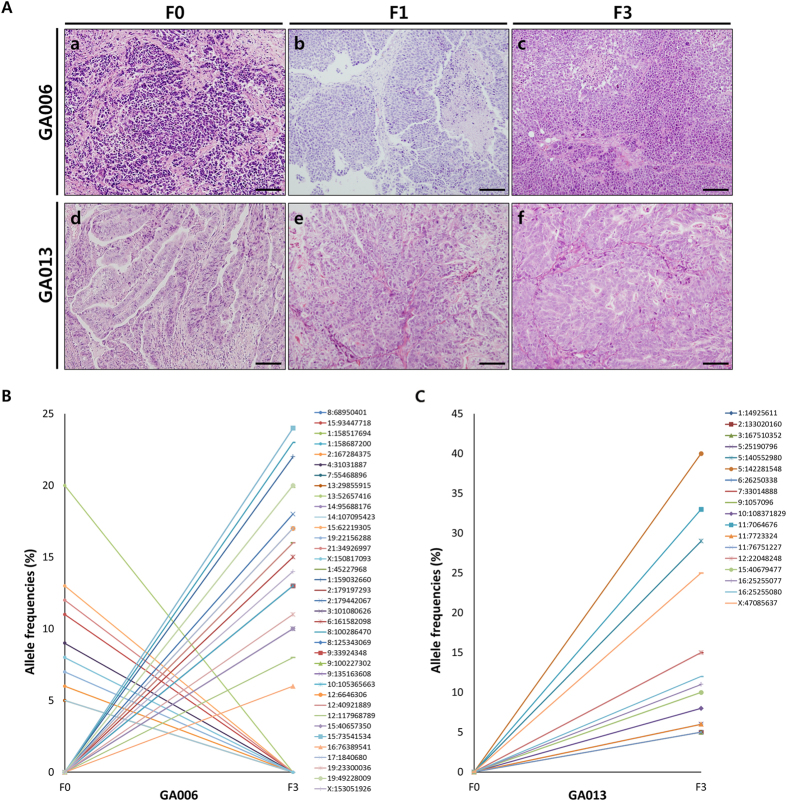
Histologic and genomic features between primary and PDX tumours. (**A**) Tumour section slides were stained to H & E for comparing histology of F1 and F3 PDX tumours with their corresponding F0 tumour in two PDX models. a, F0 from GA006; b, F1 from GA006; c, F3 from GA006; d, F0 from GA013; e, F1 from GA013; f, F3 from GA013. Scale bars = 100 μm. (**B,C**) *De novo* and disappeared mutations between F0 and F3 PDX tumours of GA006 (**B**) and GA013 (**C**) models. *De novo* mutations are defined as total allele count being zero in the F0 and greater than or equal to five in F3. The opposite cases are disappeared mutations.

Histological reviews were routinely conducted for established PDX tumours. As shown in Fig. 1A, histology (histological type and Lauren classification) of F1 and F3 PDX tumours (from two representative cases, GA006 and GA013) were well-matched with those of primary tumours. Using whole exome and RNA sequencing from primary (F0) tumours and F3 PDX tumours, these two representative cases were analysed in depth to determine whether genetic features of PDX tumours changed during rounds of subgrafting.

To assess the overall genomic changes in PDX tumours, we first investigated whole exome sequencing data between F0 and F3 tumours. There were 22 *de novo* mutations and 15 disappeared mutations in GA006, and 18 *de novo* mutations in GA013 PDX tumours (Fig. 1B,C). Many of the *de novo* and disappeared mutations were synonymous variants which unlikely to be deleterious to its protein function. In RNA sequencing analysis, the mRNA expression levels of those genes were similar between F0 and F3 (Supplementary Table S4).

**Figure 2:**
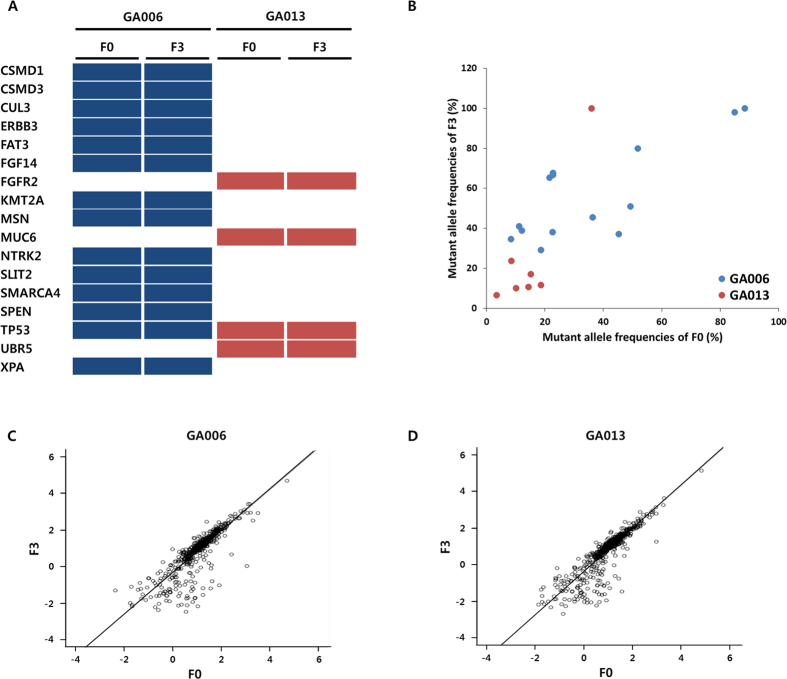
Correlation of genetic features for 726 cancer-related genes between primary and PDX tumours. (**A**) Mutations in F0 and F3 tumours among 726 cancer-related genes were indicated in GA006 (dark blue bars) and GA013 (red bars) PDX models. (**B**) Mutant allele frequencies between F0 and F3 tumours for 14 and 7 mutations detected in GA006 (blue dots) and GA013 (red dots) PDX models, respectively. (**C,D**) The correlation of mRNA expression levels for 726 cancer-related genes in F0 and F3 tumours was represented as scatter plot in GA006 (**C)** and GA013 (**D**) PDX models.

Next, to further investigate the genomic differences in cancer relevant genes between the primary and PDX tumours, we focused on 726 cancer-related genes (Supplementary Table S5), which were catalogued from COSMIC, Foundation Medicine, Oncomine Cancer Research Panel, and TCGA database. As shown in Fig. 2A, all the cancer-related genes were stable during establishment and subgrafting of F3 tumours and mRNA sequencing analysis revealed no significant differences in expression levels between F0 and F3 (Supplementary Table S6). When comparing allele frequencies between F0 and F3 tumours, correlation analyses showed that allele frequencies between F0 and F3 tumours were well correlated (r = 0.78, and r = 0.88 for GA006 and GA013, respectively, *p* < 0.001, Fig. 2B). Further, mRNA expression levels of 726 cancer-related genes were well correlated between F0 and F3 tumours (r = 0.99 and r = 0.99 for GA006 and GA013, respectively; Fig. 2C,D). In aggregate, these in-depth analyses showed that not only mutation profiles of the genes, but also RNA expression levels of corresponding genes analysed by RNA sequencing were stable over subsequent passages.

### Intra-generational comparison of PDX tumours

To address the issue of intra-generational heterogeneity of PDX tumours, additional analyses were carried out. A thorough histological assessment revealed no observable heterogeneity among individual tumours in F3 generation of the two representative PDX cases (24 and 14 cases of F3 tumours in GA006 and GA013, respectively). In whole exome sequencing analysis, some genetic variations in 726 cancer related genes were noticed across five F3 tumours of GA013 case when analysed without additional high-quality filters (Supplementary Fig. S2A). When applying high-quality filters including sequencing depth and base-call quality, intra-generational genetic variations previously observed were disappeared (Supplementary Fig. S2B). In addition, no evident discrepancy in mRNA expression was observed among the five F3 tumours of GA013 case based on a correlation analysis of the entire genes as well as 726 cancer related genes (Supplementary Fig. S2C,D).

### Lymphomatous transformation in PDX tumours

**Figure 3:**
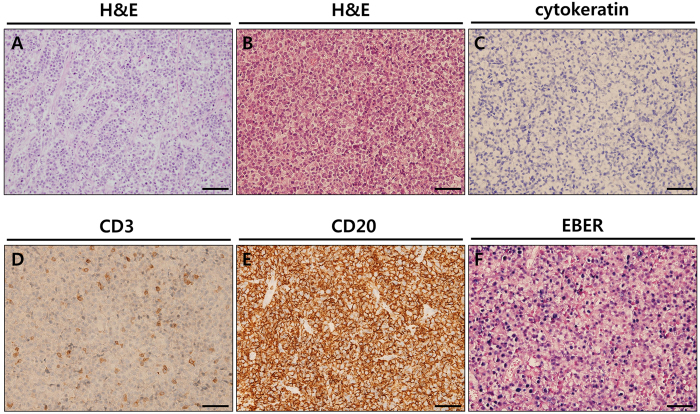
Representative histology of lymphoma transformed PDX tumours. (**A**) F0 tumour section slides were stained by H & E. (**B**) F1 tumour section slides were stained by H & E. (**C**) Cytokeratin, as a marker of epithelial tumour cells, was not detected by immunohistochemistry in F1 tumour. (**D**) CD3, as a marker of T cells, was infrequently detected by immunohistochemistry in F1 tumour. (**E**) CD20, as a marker of B cells, was detected by immunohistochemistry in F1 tumour. (**F**) Epstein-Barr encoded RNA was detected by *in situ* hybridization in F1 tumour. Scale bars = 50 μm.

After histological review of all successful cases, we found that the histology was not maintained in five cases of F1 PDX tumours (5 out of 15 successful cases). In all five cases with histological changes, tumour tissues obtained from F1 mice showed a lymphocytic dominant pattern, regardless of primary tumour histology. Immunohistochemistry (IHC) showed that these tumours were cytokeratin (CK) negative, but the cells strongly stained for CD20 and Epstein-Barr virus (EBV) early RNA (EBER) by *in situ* hybridization (ISH), indicating they were related to EBV-associated B-cell lymphoma (Fig. 3). These lymphomas were only observed in PDX tumours from the NOG mouse strain (55.6%, 5 out of 9 cases of engraftment success for the F1 NOG mice), and no lymphoma transformations were observed in six cases of PDX tumours from nude mice (F1). The detailed IHC results of these cases are summarized in Table 4. In mouse necropsy, gross tumour metastases were identified in the liver, spleen, kidney, and lymph nodes in four out of five cases of lymphoma transformation (except for GA054-1). All of metastatic tumours were confirmed as lymphomas by histologic evaluation. In contrast, there were no tumour metastases observed in PDX tumours of histologically confirmed adenocarcinomas.

**Figure 4:**
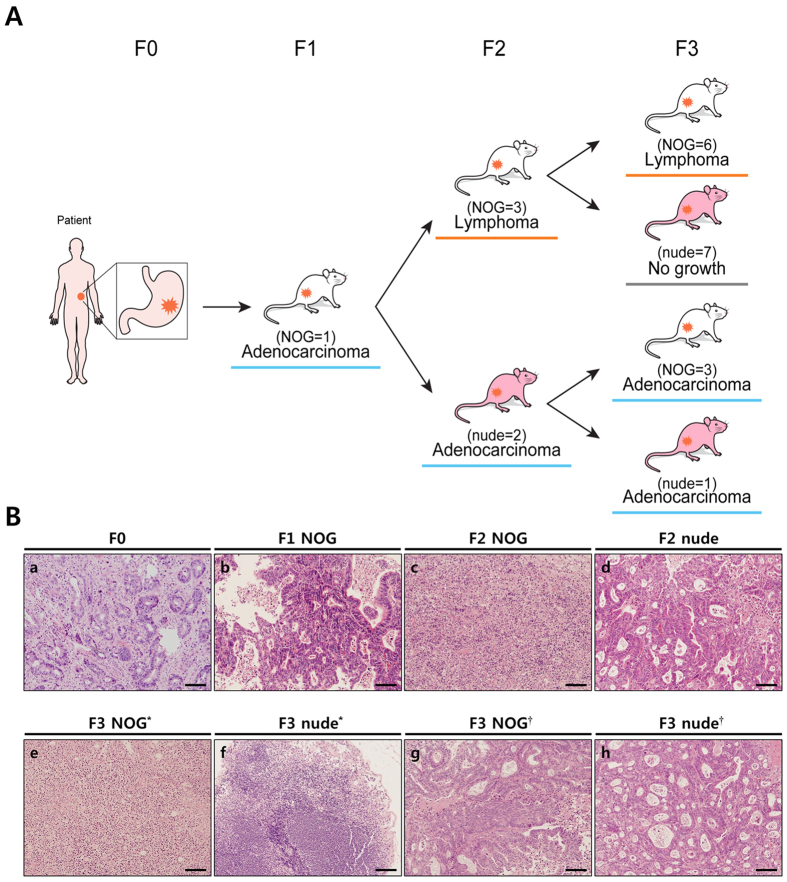
Schematic drawing of sequential histological changes into lymphomas according to the mouse strain during subpassaging in PDX model. (**A**) An engrafted adenocarcinoma in an F1 NOG mouse changed into a lymphoma in F2 NOG mice. It was retained in F3 NOG mice, but was not engrafted into F3 nude mice. However, an adenocarcinoma engrafted from an F1 NOG mouse into F2 nude mice retained their histology, and the tumour was not changed into a lymphoma in an F3 nude mouse as well as in F3 NOG mice. (**B**) Each section slide of primary and PDX tumour during subpassaging was stained by H & E for comparing their histological changes. a, adenocarcinoma in a primary tumour; b, adenocarcinoma in an F1 NOG mouse; c, lymphoma in an F2 NOG mouse; d, adenocarcinoma in an F2 nude mouse; e, lymphoma in an F3 NOG mouse; f, no tumour growth in an F3 nude mouse from an F2 NOG mouse; g, adenocarcinoma in an F3 NOG mouse from an F2 nude mouse; h, adenocarcinoma from an F3 nude mouse from an F2 nude mouse. Scale bars = 100 μm. *from F2 NOG mice, †from F2 nude mice.

Figure 4 shows an interesting case illustrating the relationship between histological changes to lymphoma and the mouse strain. The F1 tumour, which was grown in a NOG mouse showed the same histology compared to the F0 tumours. This F1 tumour was sliced and the fragments were implanted into three NOG mice and three nude mice (F2). All tumours, which were engrafted into NOG mice (F2) were transformed into lymphomas, while the tumours engrafted into nude mice (F2) retained their histology in two cases (engraftment failed in one case). The F2 lymphoma from NOG mice succeeded in the F3 generation, and the histology of the lymphoma was retained in all NOG mice (n = 7), whereas none of the lymphomas subgrafted into nude mice (n = 7) successfully formed tumours. F2 tumours from nude mice with sustained adenocarcinoma histology were implanted into four NOG mice and one nude mouse (F3). All of them were successfully engrafted, and the histological characteristics were maintained. When classifying lymphoma cases as unsuccessful and again analysing factors, which were correlated with engraftment success, the results were similar; Lauren classification (*p* = 0.022), percentage of tumour cells (*p* = 0.044), and *ex vivo* time (*p* = 0.013) correlated with engraftment success (n = 10), but overall procedure time was not statistically significant (*p* = 0.054).

## Discussion

Retaining histological and genetic features of the patient tumour is the most important aspect of PDX models. It has been reported that the histology of primary tumours (F0) was maintained through successive generations in various cancers, including gastric cancer^13^,^20^,^21^,^22^,^23^,^24^, which is consistent with the results of the present study. In addition, our sequencing analyses showed that the genetic features of PDX tumours, in terms of mutation profiles, allele frequencies, and RNA expression levels, correlated well with the corresponding tumour when histology was maintained. Consistent genetic features of PDX tumour models compared to primary tumours have been reported in other studies^13^,^22^,^23^,^25^,^26^,^27^,^28^,^29^. However, some genes were newly identified or disappeared in PDX tumours, compared to primary tumours, as reported in other cancer types using the PDX model^30^. Those differences were thought to be caused by heterogeneity of the primary tumour (the region of the primary tumour for the PDX tumour and extraction of DNA or RNA differed) and/or selection pressures arising during engraftment into different species (from human to mouse)^30^. In the present results, we demonstrated that genomic differences between PDX and primary tumours as well as among individual tumours in the same generation were insignificant, especially for well-known cancer-related genes. Thus, the PDX tumour model can constitute a paraclinical model that retains tumour characteristics of the original patient. Therefore PDX models can be used in cancer research and development of potential cancer therapeutics.

In the present study, we identified parameters correlated with engraftment success, to improve the success rate of future PDX tumour models. We found that *ex vivo* time and overall procedure time were the only significant experimental parameters affecting the success of establishing PDX tumour models for gastric cancer. Ischemic injury is closely related to viability of cells, and associated with low survival of grafts in organ transplantation^31^,^32^,^33^. Our results also indicate the importance of reducing ischemic time for successful engraftment of viable cancer cells. To the best of our knowledge, only one previous study has investigated this issue in PDX tumours, reporting that time to engraftment was not related to engraftment success^34^. However, this study used different types of tumours, and classified the time to engraftment into early (on the day of surgery or the next day) and delayed (two days after surgery) times, without additional information in detail. Other experimental factors, such as implantation site (subcutaneous vs. subrenal capsule)^13^,^18^,^35^, using Matrigel^36^,^37^, and more immunocompromised mice^18^,^38^, were reported to improve the success rate of the PDX tumour model in various cancers. However, those factors did not improve the success of PDX tumours in the present study. The possible reasons for this difference may involve the use of different types of cancers. In addition, most of the previous studies were not direct comparisons between nude and NOG strains.

Tumour aggressiveness involving metastatic tumours, lymph node metastases, and poorly differentiated histology has been reported to be related to engraftment success for colon cancer^27^,^39^. However, in the current study, tumour staging and the degree of differentiation were not related to engraftment success. Because previous studies of gastric cancer PDX tumour models reported similar results^29^,^40^, we postulate that the degree of tumour heterogeneity in gastric cancers is much higher than other reported cancers. Clinically subdominant clones in the tumour could be selected *in vivo,* giving rise to xenograft tumour formation. As high failure rates in some histologic subtypes of PDX tumour models have been reported^18^, the Lauren classification and tumour cell percentage were shown in the present study to be related to engraftment success. The diffuse type of gastric cancer was correlated with low success rate, which was consistent with a previous report^29^. In the case of diffuse type gastric cancers, tumour cells are dispersed into the stromal tissue, and the number of tumour cells is lower per unit area, making it unfavourable factor for successful engraftment. Based upon our results, the tumour cell percentage of the diffuse type was less than the intestinal or mixed/indeterminate (others) types (24.4 ± 21.4% for the diffuse type, 47.0 ± 28.7% for the intestinal type, and 41.0 ± 26.3% for others, both *p* = 0.001), which is supportive of this possibility. According to a recent study of the molecular characteristics for gastric cancer, mesenchymal-like gastric cancer comprised mostly (80.4%) of the diffuse type, by the Lauren classification, has the worst prognosis^41^. When considering that the PDX tumour is most needed for modelling tumours with poor prognoses, establishing the diffuse type gastric cancer PDX tumour model might be especially challenging.

EBV-related B-cell lymphomas in up to 68% of PDX tumour models have been recently reported in various types of cancer^37^,^42^,^43^,^44^,^45^, which used NOD/SCID^46^, NSG^47^, and NOG^48^ strains of mice. A possible explanation for the lymphoma transformation in PDX tumours might involve EBV-infected B-cells in the host (primary tumours from patients), which are latent, but can be activated because they are free from immune cell surveillance when implanted into immune-compromised mice; this is similar to the development of lymphomas in immune-compromised patients^49^. Because *H. pylori-*related gastritis is associated with gastric cancer^50^, the extent of baseline inflammation in gastric cancer is higher than in other tumours. As a result, the incidence of lymphoma in the PDX tumour model is higher in gastric cancer than other tumours^42^. In the present study, 33.3% of successful cases involved lymphoma, and all the lymphomas occurred in NOG mice (F1). Also, despite histological characteristics being retained in F1 mice, it could be changed into lymphoma in subsequent grafts if it was implanted into the NOG strain (F2). One intriguing observation is that lymphoma could not survive in nude mice. If the tumour was engrafted into nude mice, it did not change into lymphomas again in subsequent grafts, even when it was implanted into NOG mice (Fig. 4). It is possible that nude mice have natural killer cells that interfere with the reactivation of latent EBV, resulting in resistance to lymphoma transformation of the xenografted tumour^47^. It has been suggested that rituximab, a monoclonal antibody against B-cells, be used to treat mice to prevent lymphomas in the PDX tumour model^48^. However, there is no effective solution to prevent and predict EBV-related B-cell lymphomas yet. To the best of our knowledge, our study is the first to compare the results of different mouse strains using the gastric cancer PDX tumour model. We could not find any advantages of NOG mice over nude mice, and the overall success rates were not significant (10.5% for NOG mice versus 8.0% for nude mice, *p* = 0.787). Furthermore, 55.6% of the engraftment successes for NOG mice involved lymphoma, and the growth rate of the tumour was not different between NOG and nude strains in our series (Supplementary Fig. S1). Thus, we suggest that for the F1 generation, nude mice are sufficient for the gastric cancer PDX tumour model.

In summary, PDX tumours from surgical specimens of gastric cancer were successfully established in immune-compromised mice. Reducing *ex vivo* time improved the success of engraftment. Transformation to lymphoma and low success rates for diffuse type tumours were the challenges in establishing the PDX tumour model for gastric cancer. Histological and genetic similarities of the PDX model to the corresponding primary tumour demonstrated that the PDX tumour model can be translated into clinical practice for the development of effective personalized therapeutics for gastric cancer.

## Materials and Methods

### Patient selection

This study was approved by the Institutional Review Board (IRB) of Yonsei University Severance Hospital (4-2013-0526), and all patients provided written informed consent. All protocols adhered to the tenets of the Declaration of Helsinki. The samples were obtained from patients with pathologically proven adenocarcinoma of the stomach, with the following characteristics: 1) the size of the tumour was larger than 4 cm, because it should be large enough not to affect the pathological staging of the patient, 2) not only primary gastric cancer but also cancer in the remnant stomach was used; patients who were treated preoperatively with chemotherapy or chemoradiation therapy were also included, 3) if the patient received gastrectomy with metastasectomy for a metastatic lesion, both primary and metastatic tissue were obtained, and 4) patients with human immunodeficiency virus, hepatitis B, and/or hepatitis C virus were excluded.

### Tissue sampling

The tumour was obtained from a surgical specimen (F0) just after gastrectomy. A total of six pieces of tumour tissue were collected from each patient under sterile conditions. They were separated into two groups (three paired tissues in both groups): one group for the PDX tumour model in nude mice and another group for the PDX tumour model in NOG mice. In each group, there were three paired tissues: 1) tissue for the PDX tumour model was placed into RPMI 1640 medium with antibiotics (penicillin, streptomycin, gentamicin, and amphotericin B); 2) other tissue was cryopreserved in liquid nitrogen and stored at −80 °C; and 3) other tissue was fixed in 10% formalin solution for hematoxylin and eosin (H & E) staining as a representative histopathological characterisation of the implanted tumour. Each time point, such as the time of last ligation of the main artery, gastrectomy, and the beginning and completion of the PDX, was recorded to analyse the effect of processing time on engraftment success of the PDX tumour.

### Establishment of the PDX tumour model

The mice were cared according to the institutional guidelines for animal care. All animal experiments were approved by the Institutional Animal Care and Use Committee (IACUC) of the Yonsei University College of Medicine (2014-0130). The 6-week-old female athymic nude mice (Japan SLC, Inc., JAPAN) and male NOG mice (NOD/Shi-scid, IL-2 Rγnull, CIEA, JAPAN) were used for each PDX model. Before the experiments, the animals were acclimated for seven days with 12 hour light-dark cycles. The transferred tumour was placed into a sterile Petri dish containing phosphate-buffered saline (PBS), then sliced into 3 × 3 × 3 mm^3^ fragments. Typically, each fragment was implanted into a subcutaneous area in the right and left flanks. They were also implanted into the subrenal capsule (under the renal capsule) from the 16^th^ PDX case, because it was reported that subrenal capsule implantation shortened time to engraftment and improved the engraftment success^13^,^18^,^35^. Initially, Matrigel was not used, and only a single piece of tumour tissue was used for implantation. Thereafter, Matrigel-embedded tumours were tried from the 46^th^ PDX case and multiple pieces of tumours (4 ~ 6 pieces of 3 × 3 × 3 mm^3^ fragments) were tried from the 39^th^ PDX case and the number of pieces were decided according to the size of the donor tumours. The size of the implanted tumour was checked 1 ~ 3 times per week using Vernier calipers when the implanted tissue was palpable, and the volume was calculated as (length × width^2^)/2. For follow-up of subrenal capsule PDX model tumours, non-enhanced magnetic resonance imaging (MRI) [Bruker Preclinical Animal MRI (9.4 T)] was used at 1–2 month intervals. Tumour formation in the implanted site over 500 mm^3^ in size was considered as engraftment success in the case of subcutaneous implantation, and tumour formation growth (detected by MRI) was regarded as successful for subrenal implantation. For the engraftment success cases, the mouse was anaesthetised and the tumour (F1) was removed for serial transplantation to the next generation (F2, F3). After removing the tumour, the mouse was sacrificed by cervical dislocation and necropsy was done to determine possible tumour metastasis.

### Histological evaluation of primary and PDX tumours

Sections (4 μm thick) from formalin-fixed and paraffin-embedded blocks were prepared for IHC and Epstein-Barr encoded RNA *in situ* hybridization (EBER ISH). IHC was performed using a Ventana XT automated stainer (Ventana Corporation, Tucson, AZ, USA) with antibodies against CK (1:300, AE1/AE3; DAKO, Carpinteria, CA, USA), CD3 (1:100, DAKO), and CD20 (1:1600, DAKO). Every other week, there was a regular meeting with the surgeon, the pathologist, and researchers for reviewing and discussion of the histopathological characteristics of each tumour.

### Clinical and experimental information

Clinical and pathological information about enrolled patients was collected prospectively. The following variables were analysed to identify factors which were related to engraftment success of the PDX tumour model: 1) the patient’s age, patient’s sex, history of diabetes mellitus, history of hypertension, history of tuberculosis, history of smoking and alcohol, blood type, preoperative tumour markers [CEA and carbohydrate antigen (CA) 19-9], white blood cell count, percentage of neutrophils and lymphocytes, neutrophil lymphocyte ratio (NLR), and albumin level; 2) tumour pathological characteristics including size, location, gross type, tumour depth, presence of lymph node metastasis, and pTNM stage according to the 7^th^ edition of the UICC^51^, Lauren classification^19^, presence of lympho-vascular and perineural invasion, history of preoperative chemotherapy, and histology and tumour percentage in the representative tissue; and 3) experimental information including ischemic time (from last ligation of the main vessel to gastrectomy, Supplementary Fig. S3), *ex vivo* time (from gastrectomy to beginning of the PDX), the PDX time (from beginning to end of the PDX), and overall procedure time (ischemic time + *ex vivo* time + the PDX time), strain of mouse, amount of tumour and its implantation site, and whether Matrigel was used for analysis.

### Whole exome and whole RNA sequencing

For genomic analyses, two representative cases (GA006 and GA013) were selected considering two criteria: 1) PDX cases passaged greater than F3 generation at the time of analysis, 2) PDX cases representing early and advanced stage of tumours because they might have different genomic landscapes.

Genomic DNA and total RNA were extracted from the frozen tissues of primary and PDX tumour model tissues using the DNeasy Blood and Tissue Kit (Qiagen, Valencia, CA, USA) and the RNeasy Plus Mini Kit (Qiagen) according to the manufacture’s protocols. The quality of DNA was checked by 1% agarose gel electrophoresis and by the PicoGreen^®^ dsDNA Assay (Invitrogen, Carlsbad, CA, USA). For whole exome sequencing, SureSelect sequencing libraries were prepared according to the manufacturer’s instructions (Agilent Sureselect All Exon V4 kit, Santa Clara, CA, USA) using the Bravo automated liquid handler. For RNA sequencing, library preparations were performed using the Illumina^™^ Truseq RNA Access Library Prep Kit (Illumina^™^, San Diego, CA, USA) according to manufacturer’s protocols. The library qualities of both whole exome and RNA sequencing were verified by capillary electrophoresis (Bioanalyzer, Agilent). After qPCR using the SYBR Green PCR Master Mix (Applied Biosystems, Waltham, MA, USA), index-tagged libraries were combined in equimolar amounts in the pool. Cluster generation occurred in the flow cell on the cBot automated cluster generation system (Illumina^™^). The flow cell loaded on the HISEQ 2500 sequencing system (Illumina^™^) performed sequencing with 2 × 100 base pair (bp) read lengths.

### Data analyses

Continuous variables were presented with median range and analysed by the Mann-Whitney U-test. Categorical variables were presented with number and percentage and analysed by Fisher’s exact test. A *p*-value less than 0.05 was considered statistically significant. The analyses were performed by SPSS, version 19.0 software for Windows (SPSS, Chicago, IL, USA). A total of 727 cancer-related genes (Supplementary Table S5) were collected from COSMIC (Catalogue of Somatic Mutations in Cancer, n = 522), Foundation Medicine (n = 314), the Oncomine Cancer Research Panel (n = 202), and the TCGA Hotspot (n = 16), and used to compare genetic features between primary tumours (F0) and the PDX tumours (F3). We defined mutations including all sequence variants regardless of synonymous or non-synonymous alterations.

We built a new reference genome combining the human and mouse reference genomes^52^; to this reference genome, we aligned whole exome sequencing information by using Burrows–Wheeler Aligner software^53^. To avoid mouse contamination, we excluded aligned sequences from the mouse reference genomes. Then, we removed duplicated reads by using SAMtools^54^. We called somatic mutations, including single nucleotide variants and small insertions/deletions using Virmid^55^ and muTect^56^ (Supplementary Fig. S4). To determine the potential functional consequences of detected variants, we annotated them using VEP (ENSEMBL’s Variant Effect Predictor)^57^. To determine high-confidence variants, bidirectionality was included as a parameter for variants selection and all the variants called were manually inspected with Integrative Genomics Viewer (IGV). Called somatic mutations against control were further filtered based on sequencing depth and base-call quality (≥20 of high quality depth and ≥5 total allele counts)^58^,^59^ in both F0 and F3 tumours in 726 cancer related genes. To define *de novo* and disappeared mutations, mutations were filtered with ≥20 of high quality depth in both F0 and F3; if allele frequency was zero in the primary tumour but the total allele count of F3 was greater than or equal to five, it was considered *de novo* mutations, and the opposite cases were considered disappeared mutations. RNA sequencing data from the Illumina^™^ FASTQ format were assigned to human transcripts using the Xenome tool^60^. Human transcript data were then processed with the Tophat-Cufflinks pipeline. We aligned reads to the genome with TOPHAT and used Cufflinks for transcriptome assembling, and Cuffdiff script from Cufflinks was used for gene expression analysis with the -classic-fpkm option. Then, we used CummeRbund to visualize RNA sequencing analysis results.

## Additional Information

**How to cite this article**: Choi, Y. Y. *et al.* Establishment and characterisation of patient-derived xenografts as paraclinical models for gastric cancer. *Sci. Rep.* **6**, 22172; doi: 10.1038/srep22172 (2016).

## Supplementary Material

Supplementary Tables S1-S3 and Figures S1-S4

Supplementary Table S4

Supplementary Table S5

Supplementary Table S6

## Figures and Tables

**Table 1:** Summary of characteristics of established PDX models for gastric cancer.

Case No.	Age	Sex	Histology	Lauren	F1 mouse			
					% of tumour cells	Strain	Implanted site	Duration in F1 (days)†
GA001	72	F	Mixed*	Indeterminate	30	NOG	subcutaneous	101
GA003	54	M	MD	Intestinal	5	NOG	subcutaneous	94
GA006	73	M	PD	Intestinal	60	nude	subcutaneous	95
GA013	79	M	PD	Intestinal	90	nude	subcutaneous	70
GA018	78	F	PD	Intestinal	80	NOG	subrenal capsule	99
GA026	61	M	MD	Mixed	50	NOG	subcutaneous	160
GA044	67	M	MD	Intestinal	20	nude	subcutaneous	125
GA046	62	M	MD	Intestinal	90	NOG	subcutaneous	139
GA051	52	F	Mucinous	Intestinal	50	NOG	subcutaneous	70
GA052	61	F	Mucinous	Intestinal	70	nude	subcutaneous	112
GA054-1	48	M	MD	Intestinal	NA	NOG	subcutaneous	91
GA054-2	59	F	PD	Indeterminate	90	NOG	subcutaneous	48
GA058	67	M	PD	Intestinal	70	nude	subcutaneous	76
GA060	80	M	MD	Intestinal	60	nude	subcutaneous	44
GA061	54	F	MD with SRC component	Intestinal	10 (SRC only)	NOG	subcutaneous	57

^*^Adeno-neuroendocrine mixed carcinoma.

^†^Interval between implantation and subpassage to F2.

M, male; F, female; MD, moderately differentiated tubular adenocarcinoma; PD, poorly differentiated tubular adenocarcinoma; SRC, signet ring cell; NOG, NOD/Shi-scid/IL-2 Rγnull mouse; NA, not available.

**Table 2:** Pathological characteristics related to engraftment success of PDX tumours of gastric cancer.

Variables	Number (%)		*p*-value
	Successful (n = 15)	Unsuccessful (n = 47)	
**Size** (mm)†	55 (23–110)	50.0 (15–167)	0.619
**Location**			0.791
Upper	3 (20.0)	14 (29.8)	
Middle/Lower	11 (73.3)	30 (63.8)	
Others (whole, RGC)	1 (6.7)	3 (6.4)	
**Gross type**			0.321
EGC	9 (60.0)	18 (38.3)	
Borrman I/II	5 (33.3)	20 (42.6)	
Borrman III/IV	1 (6.7)	9 (19.1)	
pT stage*			>0.999
pT1/2	5 (33.3)	15 (33.3)	
pT3/4	10 (66.7)	30 (66.7)	
**LN metastasis**			0.660
negative	7 (46.7)	25 (53.2)	
positive	8 (53.3)	22 (46.8)	
pTNM stage*			0.353
I/II	8 (53.3)	30 (66.7)	
III/IV	7 (46.7)	15 (33.3)	
Histology*			0.301
Differentiated	7 (46.7)	12 (26.7)	
Undifferentiated	7 (46.7)	29 (64.4)	
Others‡	1 (6.7)	4 (8.9)	
Lauren*			**0.005**
Intestinal	12 (80.0)	20 (44.4)	
Diffuse	0 (0)	18 (40.0)	
Others (mixed & indeterminate)	3 (32.0)	7 (15.6)	
LVI*			0.554
No	7 (46.7)	26 (57.8)	
Yes	8 (53.3)	19 (42.2)	
PNI*			0.238
No	10 (66.7)	21 (46.7)	
Yes	5 (33.3)	24 (53.3)	
**Preoperative chemotherapy**			0.626
No	13 (86.7)	43 (91.5)	
Yes	2 (13.3)	4 (8.5)	
**Variables**°	**Successful (n = 15)**	**Unsuccessful (n = 97)**	*p***-value**
**% of tumour cells**†	60 (5–90)	30 (0–90)	**0.025**

^*^There was complete pathologic regression of tumours after preoperative chemotherapy in two patients.

^†^Median with range.

^‡^Lymphoepithelioma-like carcinoma (carcinoma with lymphoid stroma) and mixed adeno-neuroendocrine carcinoma.

^°^Based on the number of representative tissues.

RGC, remnant gastric cancer; EGC, early gastric cancer; LN, lymph node; LVI, lympho-vascular invasion; PNI, peri-neural invasion.

**Table 3:** Experimental factors related to engraftment success of PDX tumours of gastric cancer.

Variables	Number (%)		*p*-value
	Successful (n = 15)	Unsuccessful (n = 47)	
Ischemic time*	20 (5–45)	20 (0–70)	0.498
*Ex vivo* time*	75 (43–167)	135 (44–272)	**0.003**
PDX time*	17 (8–37)	17 (5–48)	0.895
Overall time*	123 (77–226)	167 (74–357)	**0.01**
**Tumour amount**			0.108
Single piece	8 (53.3)	36 (76.6)	
Multi-piece	7 (46.7)	11 (23.4)	
**Variables**	**Successful (n = 15)**	**Unsuccessful (n = 146)**	***p*-value**
**Strain**			0.787
nude	6 (40.0)	69 (47.3)	
NOG	9 (60.0)	77 (52.7)	
**Implanted site**			0.308
Subcutaneous	14 (93.3)	117 (80.1)	
Subrenal capsule	1 (6.7)	29 (19.9)	

^*^Minutes.

^†^Based on the number of mice.

PDX, patient derived xenograft; NOG, NOD/Shi-scid/IL-2 Rγnull mouse.

**Table 4:** Summary of cases where tumour histology was changed into EBV-related B-cell lymphoma.

Case No.	Histology	F1 mouse					
		Strain	Mirror histology	EBER	CK (AE1/AE3)	CD20	CD3
GA001	Mixed	NOG	B cell lymphocyte	Positive	Negative	Positive	Negative
GA018	PD	NOG	B cell lymphocyte	Positive	Negative	Positive	Negative
GA054-1	MD	NOG	B cell lymphocyte	Positive	Negative	Positive	Negative
GA054-2	PD	NOG	B cell lymphocyte	Positive	Negative	Positive	Positive
GA061	MD with SRC component	NOG	B cell lymphocyte	Positive	Negative	Positive	Negative

MD, moderately differentiated tubular adenocarcinoma; PD, poorly differentiated tubular adenocarcinoma; SRC, signet ring cell; NOG, NOD/Shi-scid/IL-2 Rγnull mouse; EBER, Epstein-Barr virus-encoded RNA *in situ* hybridization; CK, cytokeratin.
