# Supplementary material for: Establishment and characterisation of patient-derived xenografts as paraclinical models for gastric cancer
Source: Sci Rep. 2016 Mar 1;6:22172. doi: 10.1038/srep22172 (PMC4772087; doi:10.1038/srep22172)
Supplement: Supplementary Tables S1-S3 and Figures S1-S4 [file srep22172-s1.doc]

**Establishment and characterisation of patient-derived xenografts as paraclinical models for gastric cancer**

**Running title: PDX in gastric cancer**

**Yoon Young Choi^1,2‡^, Jae Eun Lee^1,2‡^, Hyunki Kim^3^, Moon Hee Sim^1,2^, Ka-Kyung Kim^4^, Gunho Lee^4^, Hyoung-Il Kim^1^, Ji Yeong An^1,7^, Woo Jin Hyung^1^, Choong Bae Kim^1^, Sung Hoon Noh^1,5^, Sangwoo Kim^4^ and Jae-Ho Cheong^1,2,5,6,8*^**

^1^Department of Surgery, Yonsei University College of Medicine, Seoul, Korea

, ^2^Yonsei Biomedical Research Institute, Yonsei University College of Medicine, Seoul, Korea

, ^3^Department of Pathology, Yonsei University College of Medicine, Seoul, Korea

^4^Severance Biomedical Science Institute, Yonsei University College of Medicine, Seoul, Korea

^5^Brain Korea 21 PLUS Project for Medical Science, ^6^Department of Biochemistry & Molecular Biology, Yonsei University College of Medicine, Seoul, Korea

^7^Department of Surgery, Samsung Medical Center, Sungkyunkwan University School of Medicine, Seoul 06351, Korea

^8^ Open NBI Convergence Technology Research Laboratoy of Department of Surgery, Yonsei University College of Medicine, Seoul, Korea

^*^Corresponding author: Jae-Ho Cheong, MD, PhD

Department of Surgery, Yonsei University College of Medicine,

50 Yonsei-ro, Seodaemun-gu, 120-752, Seoul, Korea

Tel: +82-2-2228-2094; Fax: +82-2-313-8289; E-mail: JHCHEONG @yuhs.ac

Keywords: patient-derived xenograft, gastric cancer, translational research

**^‡^**These authors contributed equally to this study.

**Supplementary Table S1. Baseline characteristics of the 62 enrolled patients.**

| **Variables** | **Number (%)** |
| --- | --- |
| **Age** | 61 (31-90)^†^ |
| **Sex** (M/F) | 48/14 (77.4/22.6) |
| **DM** (no/yes) | 56/6 (90.3/9.7) |
| **HTN** (no/yes) | 44/18 (71.0/29.0) |
| **Tuberculosis** (no/yes) | 57/5 (91.9/8.1) |
| **Smoking** (non/ever/current) | 36/11/15 (58.1/17.7/24.2) |
| **Alcohol** (non/ever/current) | 31/6/25 (50.0/9.7/40.3) |
| **Blood type** |  |
| A/B/O/ABO | 25/14/18/5 (40.3/22.6/29.0/8.1) |
| **CEA** (normal/high) | 59/3 (95.2/4.8) |
| **CA19-9** (normal/high) | 56/5 (91.8/8.2) |
| **WBC** | 6,265 (3,310-26,300)^†^ |
| **Neutrophil** (%) | 58.9 (26.0-85.6)^†^ |
| **Lymphocyte** (%) | 29.9 (10.1-60.0)^†^ |
| **NLR** | 1.91 (0.43-8.48)^†^ |
| **Albumin** | 4.3 (3.3-5.3)^†^ |
| **Size** (mm) | 50 (15-167)^†^ |
| **Location** |  |
| Upper | 17 (27.4) |
| Middle/Lower | 41 (66.1) |
| Others (whole, RGC) | 4 (6.5) |
| **Gross type** |  |
| EGC | 10 (16.1) |
| Borrman I/II | 27 (43.5) |
| Borrman III/IV | 25 (40.3) |
| **pT stage**^*^ |  |
| pT1/2 | 20 (32.3) |
| pT3/4 | 40 (64.5) |
| **LN metastasis** |  |
| negative | 32 (51.6) |
| positive | 30 (48.4) |
| **pTNM stage**^*^ |  |
| I/II | 38 (61.3) |
| III/IV | 23 (37.1) |
| **Histology**^*^ |  |
| Diff./Undiff./Others^‡^ | 19/36/5 (30.6/58.1/8.1) |
| **Lauren**^*^ |  |
| Intestinal/Diffuse/Others (mixed & indeterminate) | 32/18/10 (51.6/29.0/16.1) |
| **LVI** (no/yes) | 34/27 (54.8/43.5) |
| **PNI** (no/yes) | 32/29 (51.6/46.8) |
| **pre-operative chemotherapy** (no/yes) | 56/6 (90.3/9.7) |

^*^There was complete pathologic regression of tumours after preoperative chemotherapy in two patients.

^†^Median with range

^‡^Lymphoepithelioma-like carcinoma (carcinoma with lymphoid stroma) and mixed adeno-neuroendocrine carcinoma

M, male; F, female; DM, diabetes mellitus; HTN, hypertension; WBC, white blood cells; CEA, carcinoembryonic antigen; CA19-9, carbohydrate antigen 19-9; NLR, neutrophil-lymphocyte ratio (neutrophil/lymphocyte); RGC, remnant gastric cancer; EGC, early gastric cancer; LN, lymph node; Diff., differentiated; Undiff., undifferentiated type; LVI, lympho-vascular invasion; PNI, peri-neural invasion

**Supplementary Table S2. Patients’ characteristics related to engraftment success of patient-derived xenografts in gastric cancer.**

|  | **Number (%)** | |  |
| --- | --- | --- | --- |
| **Variables** | **Successful (n=15)** | **Unsuccessful (n=47)** | ***p*-value** |
| **Age**^†^ | 62 (48-80) | 59.0 (31-90) | 0.205 |
| **Sex** |  |  | 0.082 |
| Male | 9 (60.0) | 39 (83.0) |  |
| Female | 6 (40.0) | 8 (17.0) |  |
| **DM** |  |  | 0.321 |
| No | 15 (100.0) | 41 (87.2) |  |
| Yes | 0 (0) | 6 (12.8) |  |
| **HTN** |  |  | >0.999 |
| No | 11 (73.3) | 33 (70.2) |  |
| Yes | 4 (26.7) | 14 (29.8) |  |
| **Tuberculosis** |  |  | 0.323 |
| No | 15 (100.0) | 42 (89.4) |  |
| Yes | 0 (0) | 5 (10.6) |  |
| **Smoking** |  |  | 0.328 |
| None | 11 (73.3) | 25 (53.2) |  |
| Ever | 1 (6.7) | 10 (21.3) |  |
| Current | 3 (20.0) | 12 (25.5) |  |
| **Alcohol** |  |  | 0.433 |
| None | 9 (60.0) | 22 (46.8) |  |
| Ever | 0 (0) | 6 (12.8) |  |
| Current | 6 (40.0) | 19 (40.4) |  |
| **Blood type** |  |  | 0.549 |
| A | 6 (40.0) | 19 (40.4) |  |
| B | 5 (33.3) | 9 (19.1) |  |
| O | 4 (26.7) | 14 (29.8) |  |
| AB | 0 (0) | 5 (10.6) |  |
| **CEA** |  |  | 0.143 |
| Normal | 13 (86.7) | 46 (97.9) |  |
| High | 2 (13.3) | 1 (2.1) |  |
| **CA19-9** |  |  | >0.999 |
| Normal | 14 (93.3) | 43 (91.5) |  |
| High | 1 (6.7) | 4 (8.5) |  |
| **WBC**^†^ | 6000 (3720-7490) | 6330 (3310-26300) | 0.130 |
| **Neutrophil** (%)^†^ | 60.2 (33.0-68.9) | 57.9 (26.0-85.6) | 0.500 |
| **Lymphocyte** (%)^†^ | 27.4 (21.6-54.4) | 31.3 (10.1-60.0) | 0.730 |
| **NLR** (%)^†^ | 2.30 (0.61-3.08) | 1.89 (0.43-8.48) | 0.692 |
| **Albumin** (g/dL)^†^ | 4.4 (6.3-5.1) | 4.3 (3.3-5.3) | 0.628 |

^†^Median with range

DM, diabetes mellitus; HTN, hypertension; WBC, white blood cells; CEA, carcinoembryonic antigen; CA19-9, carbohydrate antigen 19-9; NLR, neutrophil-lymphocyte ratio (neutrophil/lymphocyte)

**Supplementary Table S3.** **Relationship between using Matrigel and success of PDX tumours of gastric cancer.**

|  | **Number (%)** | |  |
| --- | --- | --- | --- |
| **Variables**^*^ | **Successful (n=9)** | **Unsuccessful (n=62)** | ***p*-value** |
| **Matrigel** |  |  | >0.999 |
| No | 3 (33.3) | 24 (38.7) |  |
| Yes | 6 (66.7) | 38 (61.3) |  |

*The matrigel was used from the 46^th^ PDX lines for 17 patients with 71 mice

PDX, patient derived xenograft


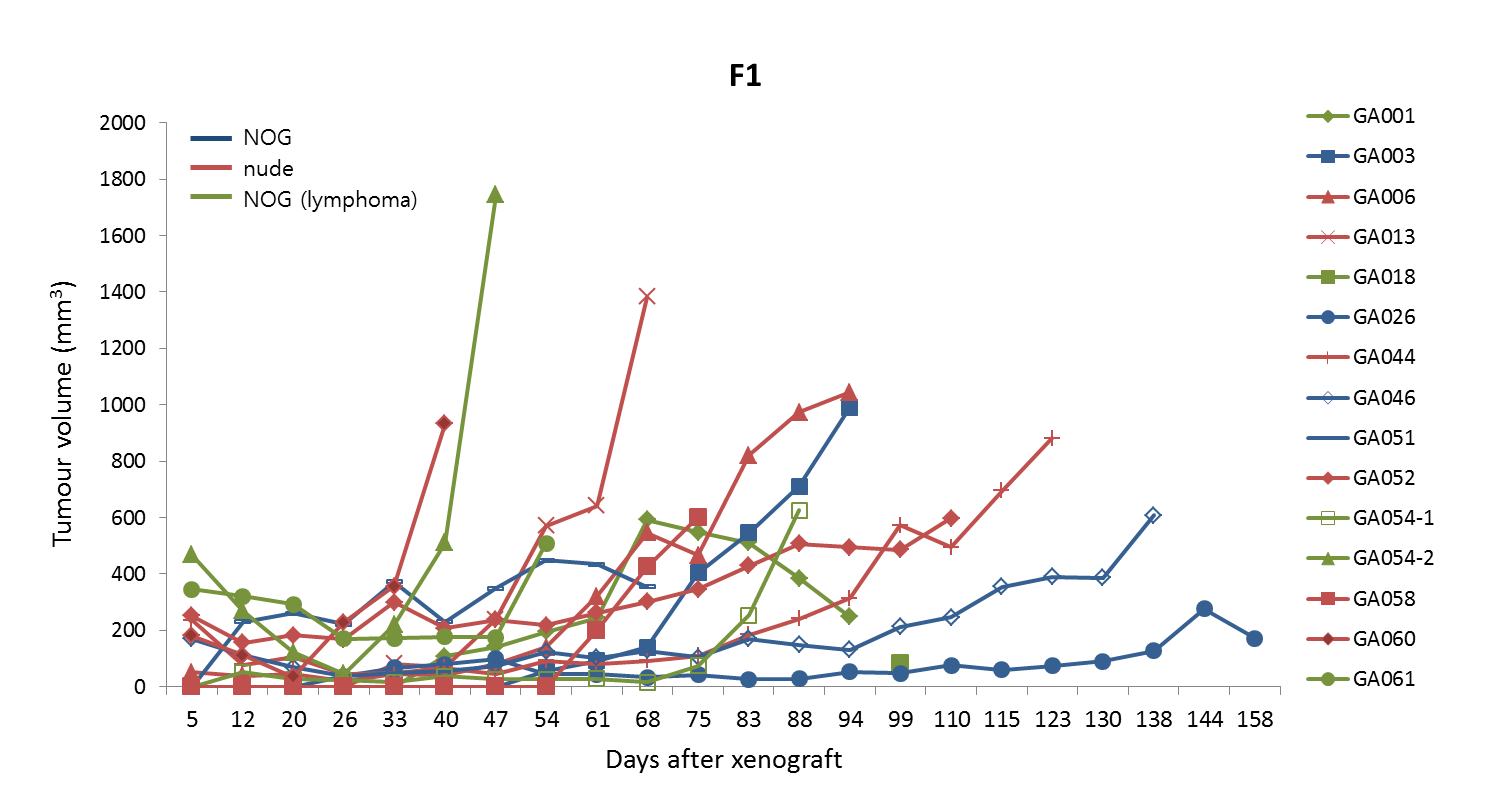


**Supplementary Figure S1. The growth rate of successful PDX tumours in F1 mice.** The size of implanted tumours was measured in 15 cases of successful PDX models. Blue line, NOG mice; red line, nude mice; green line, NOG mice with lymphoma.


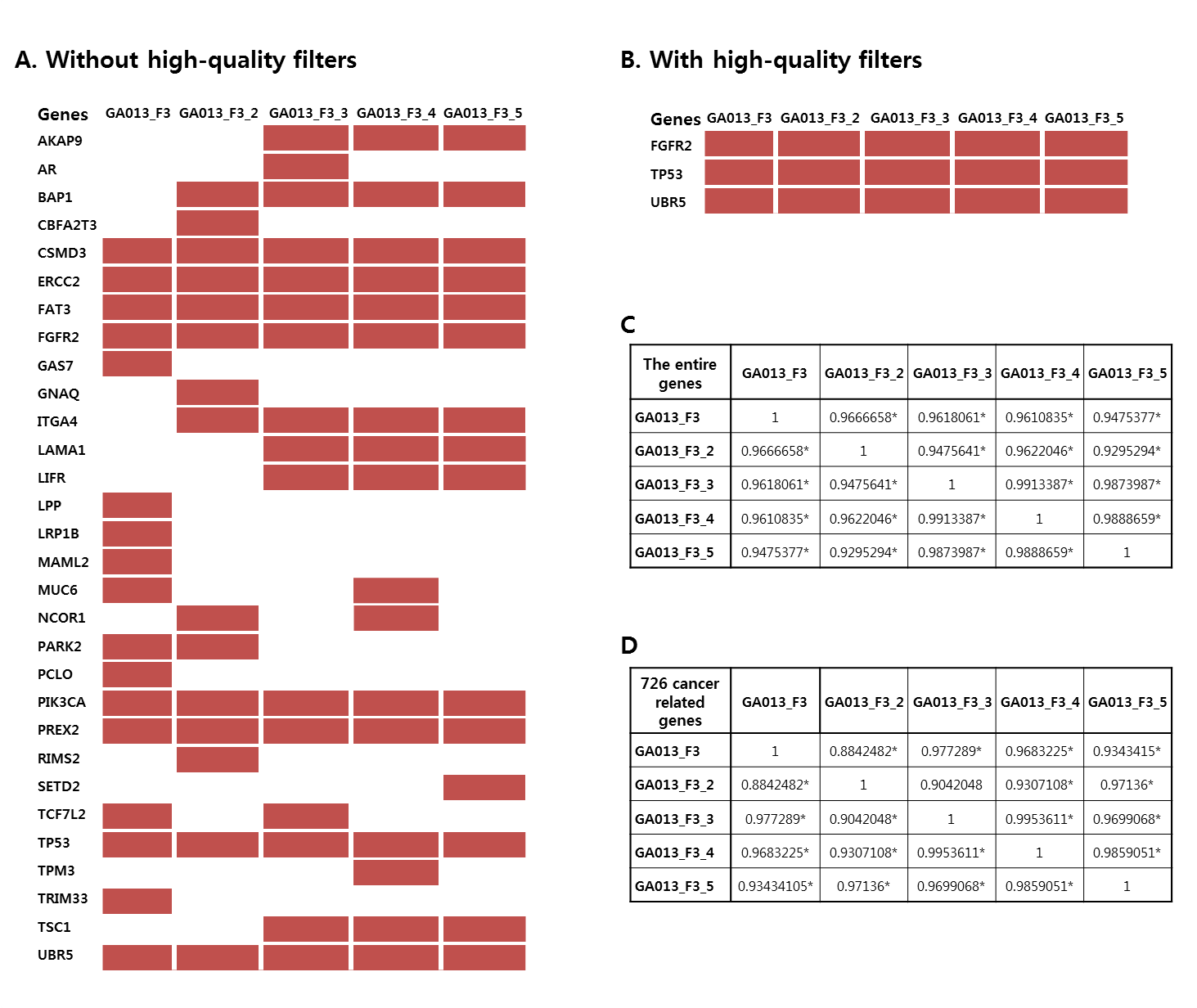


**Supplementary Figure S2. Intra-generational genomic analysis of PDX tumours.** A) Whole exome sequencing analysis without high quality filter conditions showed that there were some genetic variations in 726 cancer related genes across five F3 tumours of GA013 case. B) When applying additional high-quality filters (detailed filtering conditions were described in Supplementary Figure S4) for 726 cancer related genes, no intra-generational genomic heterogeneity was noticed. C) Whole transcriptome sequencing analysis showed that the expression levels of the entire genes were well correlated among tumours in the same generation of GA013 case. D) The mRNA expression levels of 726 cancer related genes were well correlated among individual tumours in F3 generation of GA013 case.

**p* <0.001


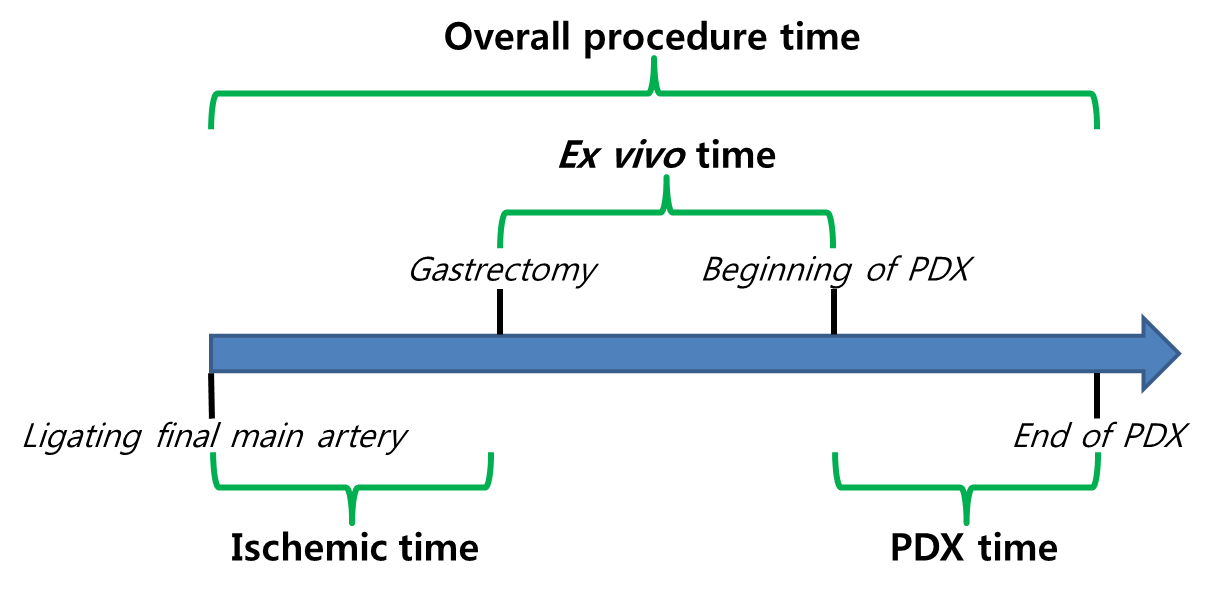


**Supplementary Figure S3. Schema depicting the procedure for establishing PDX models.** Each time point such as time of lastly ligating main artery, gastrectomy, and beginning and end of the PDX was monitored. Each indicated time (ischemic time, *ex vivo* time, PDX time, and overall procedure time) was defined according to time laps between each event.

**
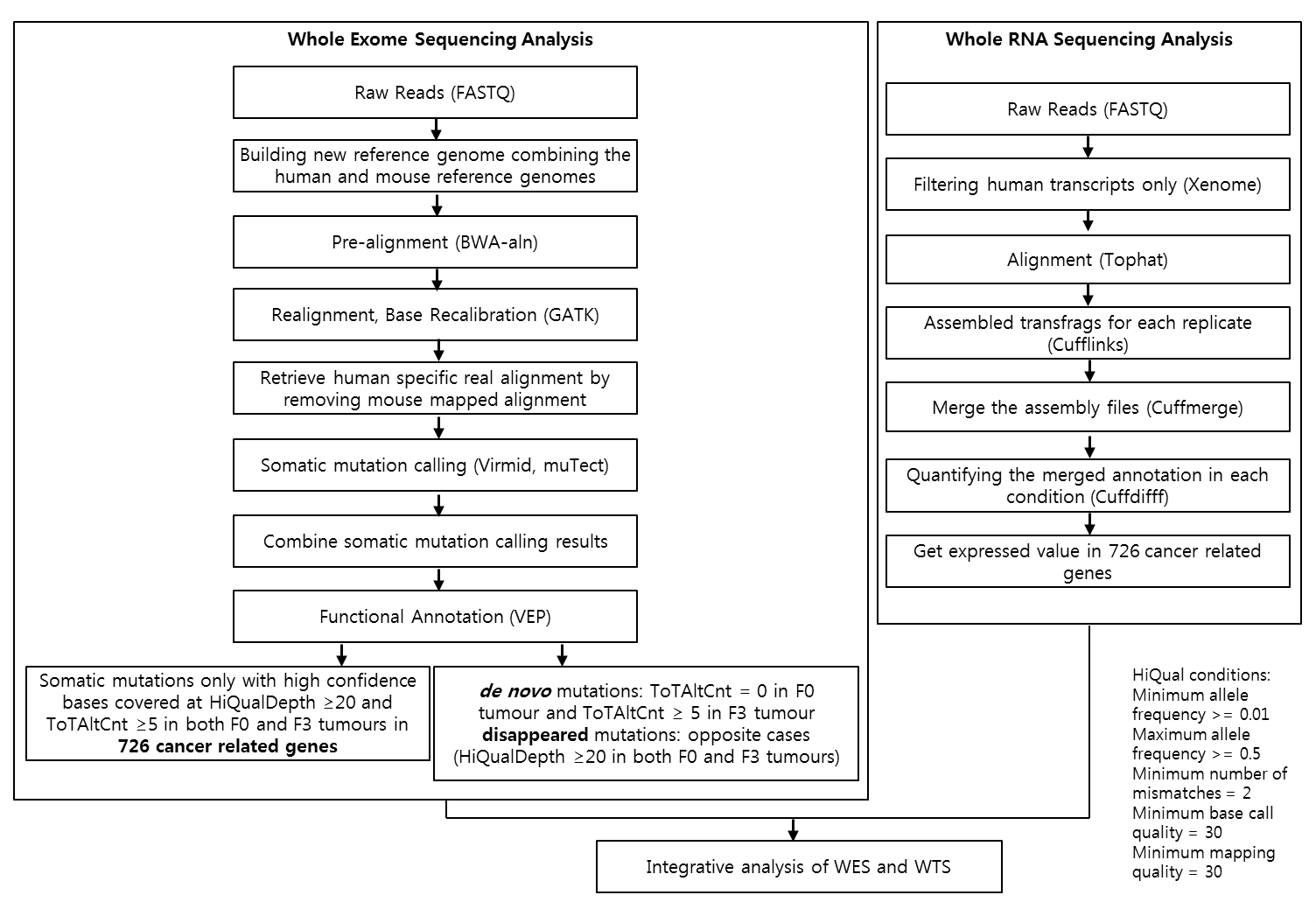
**

**Supplementary Figure S4. The work flow of whole exome sequencing and whole RNA sequencing analyses.** After removing mouse mapped alignment, somatic mutations were called using Virmid and muTect. The functional consequences of detected variants were annotated using VEP. To define *de novo*/disappeared mutations, mutations were filtered with ≥20 of high quality depth in both F0 and F3 tumours; if allele frequency was zero in the primary tumour but the total allele count of F3 was greater than or equal to five, it was considered *de novo* mutations, and the opposite cases were considered disappeared mutations. For somatic mutations in 726 cancer related genes, filters with ≥20 of high quality depth and ≥5 total allele counts in both F0 and F3 tumours were applied. For whole RNA sequencing analysis, Tophat-Cufflinks pipeline was applied.

VEP, ENSEMBL’s Variant Effect Predictor

**Supplementary Table legends**

**Supplementary Table S1. Baseline characteristics of the 62 enrolled patients.**

**Supplementary Table S2. Patients’ characteristics related to engraftment success of patient-derived xenografts in gastric cancer.**

**Supplementary Table S3.** **Relationship between using Matrigel and success of PDX tumours of gastric cancer.**

**Supplementary Figure Legends**

**Supplementary Figure S1. The growth rate of successful PDX tumours in F1 mice.** The size of implanted tumours was measured in 15 cases of successful PDX models. Blue line, NOG mice; red line, nude mice; green line, NOG mice with lymphoma.

**Supplementary Figure S2. Intra-generational genomic analysis of PDX tumours.** A) Whole exome sequencing analysis without high quality filter conditions showed that there were some genetic variations in 726 cancer related genes across five F3 tumours of GA013 case. B) When applying additional high-quality filters (detailed filtering conditions were described in Supplementary Figure S4) for 726 cancer related genes, no intra-generational genomic heterogeneity was noticed. C) Whole transcriptome sequencing analysis showed that the expression levels of the entire genes were well correlated among tumours in the same generation of GA013 case. D) The mRNA expression levels of 726 cancer related genes were well correlated among individual tumours in F3 generation of GA013 case.

**p* <0.001

**Supplementary Figure S3. Schema depicting the procedure for establishing PDX models.** Each time point such as time of lastly ligating main artery, gastrectomy, and beginning and end of the PDX was monitored. Each indicated time (ischemic time, *ex vivo* time, PDX time, and overall procedure time) was defined according to time laps between each event.

**Supplementary Figure S4. The work flow of whole exome sequencing and whole RNA sequencing analyses.** After removing mouse mapped alignment, somatic mutations were called using Virmid and muTect. The functional consequences of detected variants were annotated using VEP. To define *de novo*/disappeared mutations, mutations were filtered with ≥20 of high quality depth in both F0 and F3 tumours; if allele frequency was zero in the primary tumour but the total allele count of F3 was greater than or equal to five, it was considered *de novo* mutations, and the opposite cases were considered disappeared mutations. For somatic mutations in 726 cancer related genes, filters with ≥20 of high quality depth and ≥5 total allele counts in both F0 and F3 tumours were applied. For whole RNA sequencing analysis, Tophat-Cufflinks pipeline was applied.

VEP, ENSEMBL’s Variant Effect Predictor
